# Trend of sociodemographic and economic inequalities in the use of maternal health services in Lao *People’s Democratic Republic* from 2006 to 2017: MICS data analysis.

**DOI:** 10.1186/s41182-023-00548-2

**Published:** 2023-10-19

**Authors:** Noudéhouénou Credo Adelphe Ahissou, Daisuke Nonaka, Rie Takeuchi, Calvin de los Reyes, Manami Uehara, Phongluxa Khampheng, Sengchanh Kounnavong, Jun Kobayashi

**Affiliations:** 1 Department of Global Health, Graduate School of Health Sciences, University of the Ryukyus, Nishihara, Japan; 2 Lao Tropical and Public Health Institute, Ministry of Health, Vientiane, Lao PDR; 3 College of Arts and Sciences, University of the Philippines Manila, Manila, Philippines

**Keywords:** Free maternal and child health policy, Antenatal care, Institutional delivery, Postnatal care, Inequality, Lao PDR

## Abstract

**Background:**

Maternal mortalities remain high in the Lao *People*’*s Democratic Republic (Lao PDR)*. Since 2012, to improve access to maternal health services for all women, the country implemented several policies and strategies including user fee removal interventions for childbirth-related care. However, it remains unclear whether inequalities in access to services have reduced in the post-2012 period compared to pre-2012. Our study compared the change in sociodemographic and economic inequalities in access to maternal health services between 2006 to 2011–12 and 2011–12 to 2017.

**Methods:**

We used the three most recent Lao Social Indicator Survey datasets conducted in 2006, 2011–12, and 2017 for this analysis. We assessed wealth, area of residence, ethnicity, educational attainment, and women’s age-related inequalities in the use of at least one antenatal care (ANC) visit with skilled personnel, institutional delivery, and at least one facility-based postnatal care (PNC) visit by mothers. The magnitude of inequalities was measured using concentration curves, concentration indices (CIX), and equiplots.

**Results:**

The coverage of at least one ANC with skilled personnel increased the most between 2012 and 2017, by 37.1% in Hmong minority ethnic group women, 36.1% in women living in rural areas, 31.1%, and 28.4 in the poorest and poor, respectively. In the same period, institutional deliveries increased the most among women in the middle quintiles by 32.8%, the poor by 29.3%, and Hmong women by 30.2%. The most significant reduction in inequalities was related to area of residence between 2006 and 2012 while it was based on wealth quintiles in the period 2011–12 to 2017. Finally, in 2017, wealth-related inequalities in institutional delivery remained high, with a CIX of 0.193 which was the highest of all CIX values.

**Conclusion:**

There was a significant decline in inequalities based on the area of residence in the use of maternal health services between 2006 and 2011–12 while between 2011–12 and 2017, the largest decrease was based on wealth quintiles. Policies and strategies implemented since 2011–12 might have been successful in improving access to maternal health services in Lao PDR. Meanwhile, more attention should be given to improving the uptake of facility-based PNC visits.

## Background

Maternal mortality in low and middle-income countries has long been a major concern in development agendas [1, 2]. In 2017, over 295,000 maternal deaths occur globally from preventable and treatable causes [3]. Around 94% of all maternal deaths occur in low- and middle-income countries, mainly in sub-Saharan Africa and South Asia [4, 5]. In Southeast Asia, the Lao *People*’*s Democratic Republic* (Lao PDR) remains one of the countries with the highest numbers of maternal deaths [6, 7]. According to the Lao Social Indicator Survey of 2017, about 185 maternal deaths occur for every 100,000 live births. Meanwhile, the country has successfully decreased the incidence of maternal deaths by 78% between the years 1990 and 2015 [8]. The World Health Organization (WHO) recommends a quality continuum of care to increase the survival chances of both mothers and babies that includes a minimum of eight antenatal care (ANC) visits (initially four visits before 2016), skilled birth attendance, and four or more postnatal care (PNC) visits [9–11].

Since 2012, the government of Lao PDR introduced several policies and national strategies to improve access to basic obstetric care services to improve knowledge and access to services in vulnerable groups and subsidize direct and indirect costs to service utilization [12–14]. For instance, the 2012 adoption of the “Free Maternal and Child Health” (Free MCH) policy aimed to enhance the uptake of pregnancy-related services by addressing financial barriers through the waiving of costs related to the services [15–17]. The policy covered user fees for ANC, delivery, PNC visits, treatments, referrals, and lump sums to cover food and transportation costs, as well as incentives for mothers who request services in public health facilities in Lao PDR [16, 18]. Further fee subsidization interventions including cash transfers provided by the World Bank’s Community Nutrition Project in Lao PDR were also aimed at increasing MCH service utilization [16, 19].

Consequently, between 2012 and 2017, the use of ANC by skilled personnel increased from 54.2 to 78.4%, and the coverage of PNC has improved by 7.7% [16, 18, 20, 21]. Yet, disparities among different social subgroups persisted in access to the MCH services due to non-financial barriers such as physical accessibility, maternal education, and cultural practices [16, 18]. Furthermore, because different socio-economic groups may respond to changes differently, the anticipated increase in utilization resulting from user-fee removal interventions may not be the same across different wealth quintiles [16]. As such, the pilot phase of the Free MCH policy implementation resulted in 37% of institutional deliveries among the richest households compared to only 7% among the poorest ones [16].

Meanwhile, to date, no study has investigated the extent to which the country has progressed in reducing inequalities compared to previous periods in Lao PDR despite all efforts since 2012. Between 2000 and 2012, previous findings suggested that there was an increase in major-minor ethnic group disparities in deliveries with a professional while no change was observed in education-level related inequalities [22]. It is essential to assess the trends of inequalities in using MCH services across different periods to better identify and address persistent barriers. Therefore, this study aimed to compare the change in sociodemographic and economic inequalities in accessing ANC, institutional delivery, and PNC between 2006 to 2011–12 and 2011–12 to 2017 periods in Lao PDR.

## Methods

### Conceptual framework

**Fig. 1:**
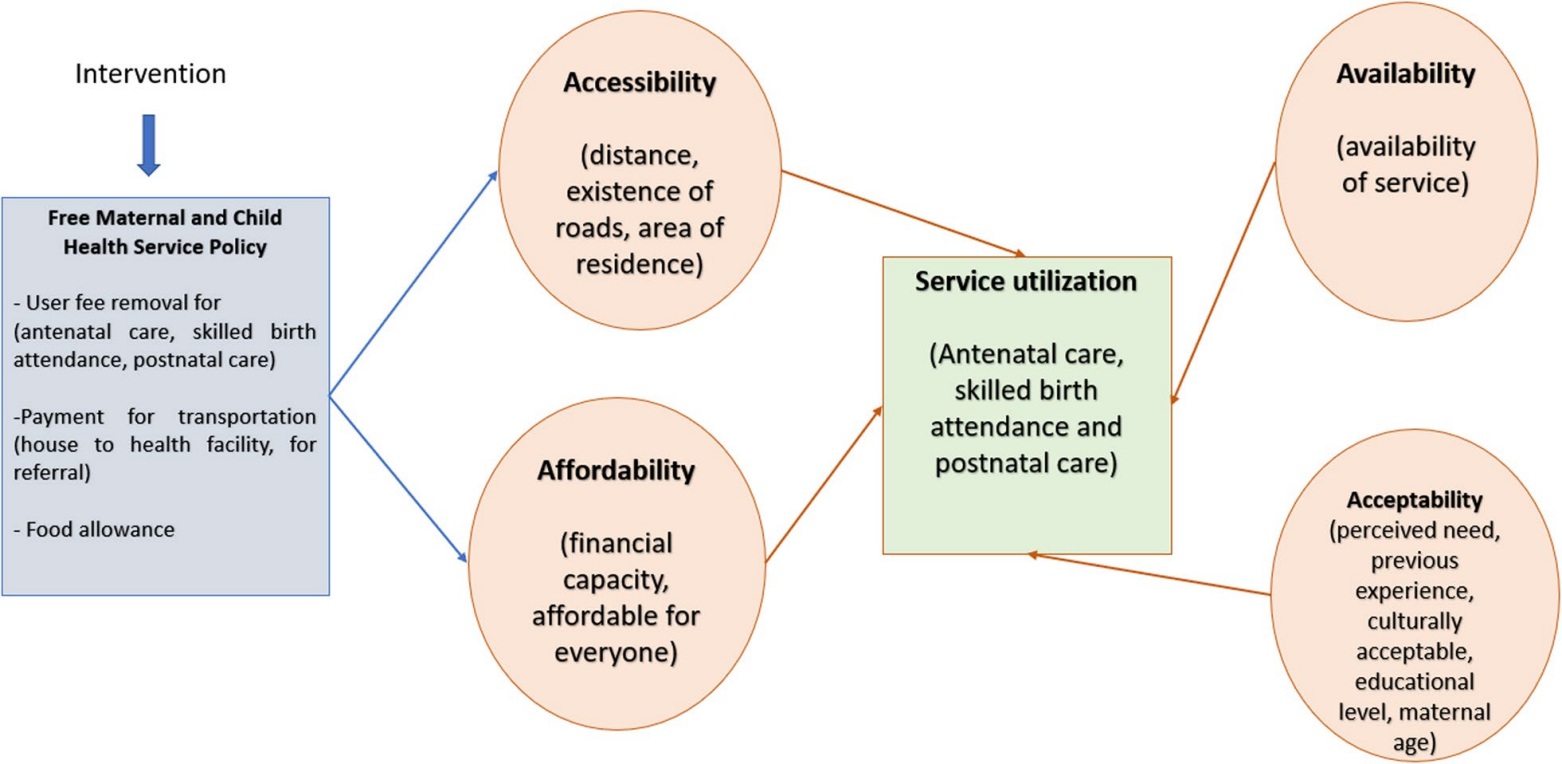
Access to maternal health service during the implementation of the Free MCH policy in Lao PDR (Source: Adapted from Levesque et al. [23], Tandon et al. [16], and Matthews et al. [58])

As shown in Fig. 1, maternal health service utilization is determined by geographical accessibility to the service provider, availability of the relevant or desired service, and affordability and acceptability of the service [23]. Therefore, the implementation of the Free MCH policy primarily seeks to address barriers in geographic accessibility to the providers (cost of transportation) and the financial barriers to service utilization (out-of-pocket payments) [16].

### Study design and data source

The study was conducted using a repeated cross-sectional design. Three of the most recent Multiple Indicator Cluster Survey (MICS) and Lao Social Indicator Survey (LSIS) datasets of Lao PDR, which are accessible in the public domain at https://mics.unicef.org/surveys, were combined (MICS 2006, LSIS I of 2011–12, and LSIS II of 2017) for the analysis. LSIS and Lao MICS are nationally representative sample surveys that collect data to assess key social development indicators in Lao PDR such as the use of maternal health services, mortality rates, use of reproductive health services, education, health nutrition, water and sanitation, child development, child protection, and rates of human immunodeficiency virus/acquired immunodeficiency syndrome.

### Study population

This study included 15–49-year-old women who had a live birth in 2 years before each survey. The women needed to reside in sampled households and agree to respond to questions related to maternal health service utilization including ANC services, birth attendance, and PNC services.

### Definitions

Existing variables in the datasets were used, whereas others were recategorized or created as necessary for our analyses. Then, all variables were harmonized across the datasets before use.

Mother’s age: we purposively recategorized women’s age at the time of the survey into four groups 15–19 years old, 20–24 years old, 25–34 years old, and 35–49 years old using an existing continuous variable of age [24]. The categorization was done to differentiate service use between adolescent women, young adult women, and older women and to avoid categories with very few women.

Educational attainment: women were grouped based on their highest education levels. Three categories were considered: “none” for women who had no formal education, and “primary” and “secondary or higher” for women whose highest educational levels were primary school and secondary school or higher, respectively.

Area of residence: according to the Lao Statistics Bureau, women were grouped depending on whether their locality is categorized as “urban”, “rural with road access”, or “rural without road access” [20, 21, 25].

Ethnicity: according to the MICS 2006, LSIS I 2011–12, and LSIS II 2017, women were asked what the ethnic group was of the heads of their households, and they were categorized as “Lao”, “Hmong”, “Khmu”, and “others” [20, 21, 25].

Wealth quintiles: these are equally sized groups generated using principal component analysis based on household ownership of 35 different assets [26]. We used this variable to approximate the socioeconomic position of the respondents’ households. Categories for this variable include “poorest”, “poor”, “middle”, “richer”, and “richest”.

### Core indicators

Three key facility-based maternal health service uses were investigated depending on how relevant they are to target services of the Free MCH policy in Lao PDR. The services include a minimum of one ANC visit provided by skilled personnel, institutional delivery, and a minimum of one facility-based PNC visit by the mothers.

ANC provided by skilled personnel: women who had a live birth in 2 years before the surveys were asked whether they saw someone for ANC during their last pregnancy. Those who reported yes were further asked whom they saw for ANC. Based on the WHO definition of skilled health personnel and as applied in the MICS, we categorized women who received ANC provided by a doctor, nurse/midwife, or auxiliary midwife as ANC from skilled personnel [27] and that from other providers such as community health workers, traditional birth attendants, relatives/friends, and others as ANC from non-skilled personnel.

Institutional delivery: we considered women with a live birth within 2 years before the surveys and who had their last deliveries in hospitals, clinics, and maternity homes to have had an institutional delivery. We categorized deliveries in individuals’ or others’ homes as non-institutional deliveries.

Mother’s facility-based postnatal visit: we grouped women into three categories: those who had at least one PNC visit in a health facility including hospitals, clinics, or maternity homes; those who received PNC visits at individuals or relatives’ homes; and those who did not receive a PNC visit. As defined in MICS, PNC visits refer to a separate visit and do not include immediate checkups provided right after the delivery while at the place of delivery. For comparison purposes, we applied a 6-week limit after delivery for the PNC visit as defined in the MICS [21]. The use of at least one facility-based PNC visit by mothers within 6 weeks of delivery was extremely low, and below 4% across most social subgroups in 2017. Therefore, the assessment of inequalities was done only for ANC and institutional delivery.

### Data analysis

Data analyses were conducted using Stata (V.17, StataCorp). We conducted a descriptive analysis to understand the socio-demographic characteristics of the study respondents and their use of maternal health services in 2006, 2011–12, and 2017. Chi-square tests were performed to compare the use of maternal health services between 2006 and 2017, and p-values with a 95% confidence interval (CI) were reported. Two of the commonly used summary measures of inequality were adopted in the present study including concentration curves and concentration indices (CIX), and equiplots. A concentration curve plots the cumulative sample population, ranked by a socioeconomic predictor variable with natural ordering such as wealth or education, against the cumulative proportion of health service utilization [28, 29]. The diagonal line from the origin also referred to as the equality line, reflects perfect equality in service utilization. For inequality measurement based on wealth, concentration curves lying below the diagonal indicate service disparities favoring the richest women while concentration curves above the equality line indicate service disparities in favor of the poorest. Similarly, in terms of educational attainment, concentration curves below the equality line show that services benefit women with secondary or higher education whereas service disparities are in favor of women with no education if the curves lie above the equality line. The CIX is calculated as twice the area between the concentration curve and the line of equality (diagonal) and measures the extent of inequality systematically associated with wealth [28, 29]. The index takes a value between − 1 and 1, with 0 indicating perfect equity. Stata commands used to generate the concentration curve plots and CIX are presented in a do-file attached as supplementary. We used equiplots to summarize absolute inequalities in access to services based on age groups, ethnicity, and area of residence as the use of concentration curves with those factors is not common in the literature [30]. Equiplots present coverage of maternal health service utilization by groups, which allows observation of levels of coverage and gaps between groups [31]. We used the *svyset* command to adjust for clustering, stratification, and the sampling weights of individual women in all analyses. Then, we displayed the absolute values of the CIX in graphs to analyze the trend in the magnitudes of socio-demographic and economic inequalities between 2006 and 2017.

### Missing data

Of 10,526 women who had a live birth in the past 2 years before the surveys, 17 (0.2%) and 51 (0.5%) women had missing information for educational attainment and ethnicity, respectively. Regarding maternal health service utilization, 113 (1.1%) of the 10,526 women had missing data for the use of at least one ANC with skilled personnel as they did not answer about whether, and from whom, they received ANC, whereas 88 (0.83%) women did not answer about whether they had an institutional delivery. Moreover, of 8904 women in the MICS 2011–12 and LSIS II 2017 who were asked about PNC visits, 124 (1.4%) had missing data for using at least one facility-based PNC visit as they failed to answer about whether, when, and where they made a PNC visit. In each analysis, women were only excluded for sociodemographic and economic factors and maternal health services for which data were missing. Finally, all women in the MICS 2006 were excluded from PNC visit analyses as they were not asked questions regarding PNC.

## Results

### Socio-demographics of participants

Table 1 shows the sociodemographic and economic characteristics of the respondents. Overall, about 26.0% (10,526) of all women of reproductive age (15–49 years) in the three datasets combined reported giving birth in 2 years before the surveys: 1622 women in MICS 2006, 4444 women in LSIS I of 2011–12, and 4460 in LSIS II of 2017. The average age of the respondents was 29 years old with a standard deviation of 9.8. Women with a primary educational level were the most dominant (40.0%) among the study respondents. Over 60.0% of women were living in a rural place with a road; most women were Lao (51.6%).

**Table 1:** Sociodemographic and economic characteristics of women

Variables	Combined dataset (N = 10,526)		MICS 2006 (N = 1622)			LSIS 2011–12 (N = 4444)			LSIS 2017 (N = 4460)			P-value^¶^
	%^a^	95% CI^b^	n	%^a^	95% CI^b^	n	%^a^	95% CI^b^	n	%^a^	95% CI^b^	
Mother’s age			**n = 1622**			**n = 4444**			**n = 4460**			0.012
15–19	11.9	11.2–12.6	188	11.8	10.1–13.8	580	12.2	11.2–13.3	557	11.5	10.5–12.7	
20–24	29.7	28.7–30.7	451	27.5	25.2–30.0	1386	30.5	28.9–32.1	1371	29.6	28.1–31.1	
25–34	45.4	44.3–46.5	739	46.7	43.8–49.6	1869	43.3	41.7–45.0	2038	47.1	4.5–48.8	
35–49	13.1	12.4–13.8	244	14.0	12.4–15.7	609	14.0	12.9–15.3	494	11.8	10.7–13.0	
Age	**Mean (SD)**		**Mean (SD)**			**Mean (SD)**			**Mean (SD)**			
	29.6 (9.8)		29.2 (9.7)			29.6 (9.9)			29.9 (9.6)			
Educational attainment			**n = 1605**			**n = 4444**			**n = 4460**			< 0.001
No education	26.1	24.5–27.8	618	39.2	34.2–44.4	1363	29.0	26.4–31.7	870	18.5	16.6–205	
Primary	40.0	38.6–41.4	727	43.2	39.3–47.3	1855	41.0	38.8–43.1	1682	37.8	35.8–39.7	
Secondary+	34.0	32.4–35.6	260	17.6	14.5–21.2	1226	30.1	27.8–32.4	1908	43.8	41.5–46.1	
Area of residence			**n = 1622**			**n = 4444**			**n = 4460**			< 0.001
Urban	23.4	21.4–25.5	238	16.3	12.5–21.1	890	22.2	19.3–25.4	1194	27.2	24.3–30.2	
Rural with road	62.8	60.3–65.3	985	54.8	47.7–61.8	3067	68.0	64.3–71.5	2673	60.4	56.9–63.8	
Rural without road	13.8	12.0–15.8	399	28.9	22.3–36.5	487	9.8	7.6–12.5	593	12.4	10.2–15.1	
Ethnicity			**n = 1621**			**n = 4438**			**n = 4416**			< 0.001
Lao	16.8	14.9–18.8	781	47.9	41.8–54.2	1776	48.0	44.7–51.4	2095	55.9	52.9–58.8	
Khmu	13.6	12.1–15.4	230	14.4	10.9–18.7	591	11.7	10.1–13.6	1286	25.7	23.1–28.5	
Hmong	18.1	16.5–19.7	230	15.6	11.1–21.4	569	12.1	9.8–14.7	831	14.4	12.2–16.9	
Other	51.6	49.3–53.8	380	22.1	16.5–28.9	1502	28.2	25.0–31.7	204	3.0	2.3–4.0	
Wealth quintiles			**n = 1622**			**n = 4444**			**n = 4460**			0.048
Poorest	27.3	25.6–29.0	516	31.7	27.3–36.4	1367	27.4	24.9–30.0	1260	25.6	23.2–28.1	
Poor	21.9	20.7–23.1	396	24.1	20.9–27.6	1010	21.5	19.9–23.2	1056	21.4	19.6–23.2	
Middle	18.7	17.6–19.8	322	18.2	15.6–21.2	848	18.8	17.2–20.5	833	18.7	17.2–20.3	
Rich	16.3	15.3–17.4	221	13.4	11.0–16.2	668	16.4	14.8–18.2	688	17.3	15.7–19.0	
Richest	15.9	14.6–17.3	167	12.6	9.8–16.1	551	15.9	14.2–17.8	623	17.1	15.4–119.0	

*SD* standard deviation

^¶^Chi-square p-value for participants comparison across datasets

^a^Column percentages

^b^95% confidence interval

### Trends of maternal health service utilization between 2006 to 2011–12 and 2011–12 to 2017 in Lao PDR

Tables 2, 3, and 4 show the coverages of the use of ANC visit with skilled personnel, institutional delivery, and mother’s facility-based postnatal visit within 6 weeks of delivery by sociodemographic and economic characteristics of women between the years 2006 and 2017 in Lao PDR.

**Table 2:** Use of at least one antenatal care (ANC) from skilled personnel by sociodemographic and economic factors in the years 2006, 2011–12, and 2017

Variables	Year 2006 (N = 1622)			Year 2011–12 (N = 4444)			Year 2017 (N = 4460)			% change between years	
	n	%^a^	95% CI^b^	n	%^a^	95% CI^b^	n	%^a^	95% CI^b^	Years 2011–12 and 2006	Years 2017 and 2011–12
Overall	**n = 1597**			**n = 4357**			**n = 4459**				
	547	35.7	31.3–40.3	2295	55.4	52.6–58.1	3438	78.4	76.1–80.5	19.7	23.0
Mother’s age	**n = 1597**			**n = 4357**			**n = 4459**				
15–19	70	38.3	30.3–47.1	285	49.7	45.0–54.4	406	74.4	69.4–78.8	11.4	24.7
20–24	167	38.7	32.9–44.9	756	58.8	55.0–62.5	1075	79.4	76.5–82.0	20.1	20.6
25–34	248	35.3	29.8–41.2	1016	57.9	54.3–61.4	1627	80.8	78.2–83.1	22.6	22.9
35–49	62	28.3	21.8–35.9	238	44.8	39.8–49.9	330	70.3	65.0–75.0	16.5	25.5
Educational attainment	**n = 1580**			**n = 4357**			**n = 4459**				
No education	93	14.5	10.7–19.4	307	23.7	20.6–27.2	431	49.5	44.5–54.6	9.2	25.8
Primary	265	38.3	33.2–43.7	991	57.3	54.0–60.5	1256	76.5	73.6–79.2	19.0	19.2
Secondary or higher	185	76.5	69.6–82.2	997	83	80.1–85-5	1751	92.2	90.5–93.6	6.5	9.2
Area of residence	**n = 1597**			**n = 4357**			**n = 4459**				
Rural without road	56	14.4	9.8–20.8	84	19.3	13.8–26.2	328	55.4	48.0–62.6	4.9	36.1
Rural with road	321	34.3	29.0–40.1	1477	50.8	47.6–53.9	2012	76.4	73.5–79.1	16.5	25.6
Urban	170	77.8	68.8–84.9	734	85.4	80.8–89.0	1098	93.3	91.0–95.1	7.6	7.9
Ethnicity	**n = 1596**			**n = 4351**			**n = 4459**				
Other	82	21.1	15.5–28.1	597	41.0	36.6–45.5	139	60.4	50.0–69.9	19.9	19.4
Hmong	24	11.2	7.1–17.2	130	24.2	19.4–29.7	537	61.3	55.3–66.9	13.0	37.1
Khmu	72	31.3	21.4–43.2	249	43.6	37.6–49.9	888	66.8	62.2–71.2	12.3	23.2
Lao	369	51.7	45.3–58.0	1314	74.5	70.4–78.2	1874	89.4	87.0–91.4	22.8	14.9
Wealth quintiles	**n = 1597**			**n = 4357**			**n = 4459**				
Poorest	86	16.6	12.2–22.2	297	23.4	20.3–26.8	659	51.8	47.3–56.2	6.8	28.4
Poor	106	24.9	19.6–30.9	434	42.9	38.7–47.1	791	74	70.4–77.4	18.0	31.1
Middle	100	31.7	25.4–38.7	536	63.4	59.0–67.5	734	88.3	85.3–90.7	31.7	24.9
Rich	115	55.3	47.4–62.8	516	78.6	74.3–82.3	645	93.7	91.3–95.5	23.3	15.1
Richest	140	88.9	82.1–93.3	512	93.7	90.4–95.9	609	97.3	94.9–98.6	4.8	3.6

^a^%: prevalence

^b^95% confidence interval

**Table 3:** Institutional deliveries by sociodemographic and economic factors in years 2006, 2011–12 and 2017

Variables	Year 2006 (N = 1622)			Year 2011–12 (N = 4444)			Year 2017 (N = 4460)			% change between years	
	n	%^a^	95% CI^b^	n	%^a^	95% CI^b^	n	%^a^	95% CI^b^	Years 2011–12 and 2006	Years 2017 and 2011–12
Overall	**N = 1597**			**N = 4381**			**N = 4460**				
	337	23.1	19.3–27.3	1645	40.7	38.1–43.4	2843	65.5	63.1–67.9	17.6	24.8
Mother’s age	**n = 1597**			**n = 4381**			**n = 4460**				
15–19	46	25.1	18.0–33.9	232	41.6	36.9–46.4	321	57.8	52.1–63.2	16.5	16.2
20–24	98	23.7	18.7–29.4	522	41.9	38.5–45.5	892	66.8	63.6–69.8	18.2	24.9
25–34	152	23.3	18.5–28.9	715	41.8	38.4–45.3	1346	67.6	56.4–66.8	18.5	25.8
35–49	41	19.1	13.7–25.9	176	33.8	29.2–38.8	284	61.8	63.1–67.9	14.7	28.0
Educational attainment	**n = 1580**			**n = 4381**			**n = 4460**				
No education	80	11.9	0.7–18.7	265	20.8	17.5–24.7	333	38.2	33.4–43.2	8.9	17.4
Primary	117	17.7	14.3–21.7	554	32.7	29.7–35.8	957	58.5	55.1–61.8	15.0	25.8
Secondary or higher	135	60.4	51.9–68.4	826	70.7	67.3–73.8	1553	83.2	80.7–85.4	10.3	12.5
Area of residence	**n = 1597**			**n = 4381**			**n = 4460**				
Rural without road	40	10.1	4.6–20.8	94	17.6	12.1–24.2	235	40.1	33.3–47.3	7.5	22.5
Rural with road	170	17.9	14.3–22.1	911	32.4	29.5–35.4	1579	60.2	56.9–63.4	14.5	27.8
Urban	127	63.6	53.0–73.0	640	76.5	71.8–80.7	1029	89.0	86.4–91.1	12.9	12.5
Ethnicity	**n = 1596**			**n = 4375**			**n = 4460**				
Other	90	22.1	13.4–34.3	488	34.3	30.2–38.6	110	46.5	37.1–56.3	12.2	12.2
Hmong	20	9.7	6.1–15.0	90	16.8	13.2–21.2	420	47.0	40.8–53.3	7.1	30.2
Khmu	26	10.9	6.8–17.0	134	23.6	19.2–28.1	662	50.5	46.1–54.8	12.7	26.9
Lao	201	31.5	25.8–37.7	930	54.7	50.5–58.9	1651	78.6	75.8–81.1	23.2	23.9
Wealth quintiles	**n = 1597**			**n = 4381**			**n = 4460**				
Poorest	75	12.8	7.5–21.1	205	15.6	12.6–19.1	452	35.5	31.4–39.8	2.8	19.9
Poor	53	13.6	9.9–18.5	236	24.4	20.8–28.3	575	53.7	49.7–57.7	10.8	29.3
Middle	39	12.3	8.6–17.2	343	40.3	36.2–44.6	623	73.1	69.0–76.7	28.0	32.8
Rich	55	29.0	22.3–36.7	378	57.6	52.8–62.3	592	86.0	82.6–88.9	28.6	28.4
Richest	115	75.9	66.4–83.3	483	89.0	85.4–91.8	601	96.3	94.2–97.6	13.1	7.3

^a^%: prevalence

^b^95% confidence interval

**Table 4:** Mother’s facility-based postnatal visit within 6 weeks of delivery by sociodemographic and economic factors in the years 2011–12, and 2017

Variables	Year 2011–12 (N = 4318)			Year 2017 (N = 4451)			% change between years 2017 and 2011–12
	n	%^a^	95% CI^b^	n	%^a^	95% CI^b^	
Overall	**n = 4357**			**n = 4459**			
	73	2.3	1.8–3.0	123	3.7	2.3–4.5	1.4
Mother’s age	**n = 4324**			**n = 4451**			
15–19	7	1.5	0.7–3.2	10	2.2	1.1–4.2	0.7
20–24	21	2.2	1.4–3.4	28	2.7	1.8–4.1	0.6
25–34	35	2.7	1.8–3.9	69	4.6	3.5–6.0	1.9
35–49	10	2.3	1.1–4.6	16	3.9	2.3–6.7	1.7
Educational attainment	**n = 4324**			**n = 4451**			
None	6	0.5	0.2–1.3	6	0.7	0.3–1.6	0.1
Primary	21	1.5	0.9–2.3	43	3.4	2.4–4.8	1.9
Secondary or higher	46	0.5	3.8–7.1	74	5.2	4.0–6.6	4.7
Area of residence	**n = 4381**			**n = 4451**			
Urban	34	5.6	3.8–8.1	52	6.3	4.6–8.4	0.7
Rural with road	37	1.5	1.1–2.2	61	2.7	2.0–3.6	1.2
Rural without road	2	0.4	0.0009–1.6	10	2.8	1.1–6.7	2.4
Ethnicity	**n = 4313**			**4407**			
Lao	54	3.9	2.9–5.3	92	5.0	4.3–6.8	1.5
Khmu	3	0.7	0.2–2.2	17	2.0	1.0–2.7	0.9
Hmong	4	0.9	0.4–2.4	9	1.0	0.5–2.4	0.1
Other	12	0.9	0.5–1.8	4	2.0	0.9–6.9	1.6
Wealth quintiles	**n = 4318**			**n = 4451**			
Poorest	8	0.7	0.3–1.4	16	1.1	0.7–2.4	0.5
Poor	7	1.0	0.5–2.3	15	1.5	0.9–2.5	0.4
Middle	14	1.9	1.0–3.4	17	2.6	1.4–5.0	0.8
Rich	15	3.0	1.8–5.1	29	4.8	3.2–7.1	1.8
Richest	29	6.8	4.6–10.0	46	9.9	7.5–13.1	3.1

^a^%: prevalence

^b^95% confidence interval

The overall coverage of at least one ANC visit from skilled personnel rose by 42.7% (from 35.7 to 78.4%) between 2006 and 2017, with almost 54.0% of that increase occurring between 2011–12 and 2017. Across all categories of sociodemographic and economic characteristics of interest, there was an increase in the uptake of ANC provided by skilled personnel. At least 70.3% of women were using ANC services provided by skilled personnel by the year 2017 regardless of age categories, whereas women in each ethnicity category had a minimum of 60.4% ANC use. In 2017, the uptake of ANC was the highest among women with a secondary educational level or higher (92.2%; 95% CI 90.5–93.6), women living in urban areas (93.3%; 95% CI 91.0–95.1), Lao women (89.4%; 95% CI 87.0–91.4), and women from the richest wealth quintile (97.3%; 95% CI 94.9–98.6). Meanwhile, the highest increases in ANC coverage were observed between the years 2011–12 and 2017 among the least favored subgroups. Hmong women, women living in rural areas, and poor women experienced increases of 37.1%, 36.1%, and 31.1%, respectively. The increase was twice as high for Hmong compared to Lao women, four times higher for rural women compared to women in urban areas, and eight times higher for the poor compared to the richest.

Similarly, to ANC coverage, the institutional delivery has steadily improved between 2006 and 2017 across all social subgroups. Over 58.0% of the total increase in institutional deliveries in that period occurred from the year 2011–12. The biggest gap (> 60%) in institutional delivery levels was among the poorest and the richest women. Regarding ethnic groups, although the Hmong minority group had the highest increase of 30.2% in institutional delivery between 2011–12 and 2017, the coverage remains 31.6% lower than that of Lao women in 2017.

Finally, unlike the use of ANC and institutional delivery, the overall coverage of PNC only increased by 1.4% in 5 years and remained below 4.0% (3.7%; 95% CI 3.0–4.5) in 2017. In 2017, the highest increase in PNC use was 4.7% among women with secondary or higher education levels, while the largest coverage was only 9.9% among the richest women.

### Trends and magnitude of sociodemographic and economic inequalities in ANC use and institutional delivery in Lao PDR between the years 2011–12 and 2017

**Fig. 2:**
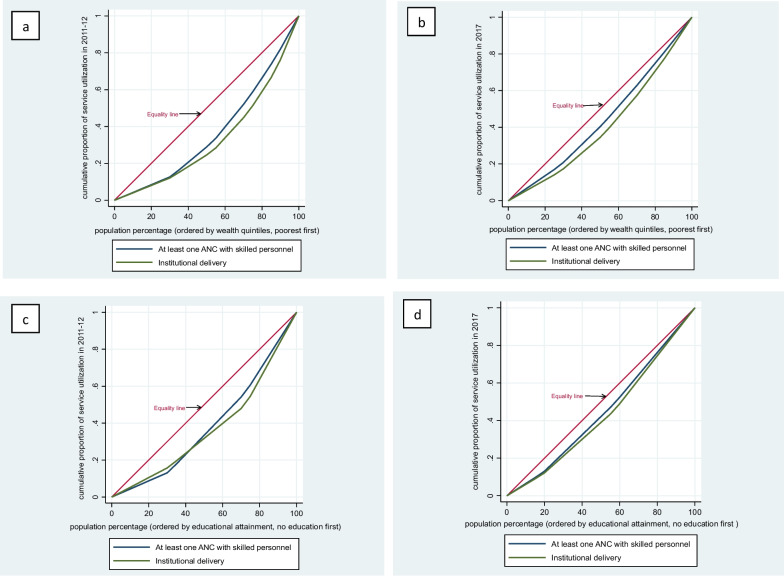
Concentration curves based on wealth and educational attainment for years 2011–12 and 2017

Figure 2 shows the concentration curves of maternal health service use by wealth quintiles and educational attainment for the years 2011–12 and 2017. Overall, there is a gap between all concentration curves and equality lines as shown in graphs depicting the existence of inequalities in the use of ANC with skilled personnel and facility-based delivery. All concentration curves lie below the equity lines as maternal health service use benefited more of the richest women and women with senior or higher education levels in both years 2011–12 and 2017. Meanwhile, concentration curves for all factors are closer to the equality line in 2017 than in 2011–12, indicating that inequalities based on sociodemographic and economic factors have decreased within the period.

**Fig. 3:**
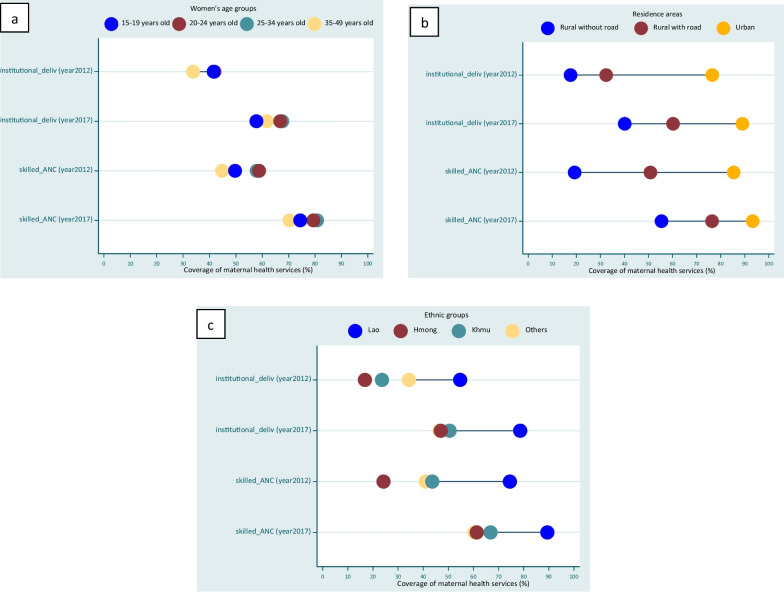
Equiplots for maternal health service utilization based on residence areas, ethnicity, and women’s age groups between the years 2011–12 and 2017

Figure 3 presents the equiplots for maternal health service utilization based on the area of residence, ethnicity, and women’s age groups between the years 2011–12 and 2017. In 2017, compared to 2011–12, while disparities persisted, the service coverage gaps decreased among extreme social groups for both ANC uptake and facility-based delivery. The gap in ANC use reduced from 66.1 to 37.9% between rural and urban women, and from 50.3 to 28.1% among Lao women and the Hmong. Similarly, the gap in institutional deliveries reduced from 58.9 to 48.9 between urban and rural women, and from 37.9 to 31.6% among Lao women and the Hmong. On the other hand, women’s age-related inequalities were almost non-existent.

Table 5 shows the magnitude of inequalities between 2006, 2011–12, and 2017 using CIX. All CIXs are different than zero, which confirms the existence of inequalities in service utilization as found earlier. In 2017, the mean of CIX values was lower for ANC compared to institutional deliveries (0.076, SD = 0.047 vs 0.115, SD = 0.067; p = 0. 011). This suggests that inequality levels were significantly lower for the use of at least one ANC visit with skilled personnel than for institutional delivery in 2017.

**Table 5:** Concentration indices (CIX) for maternal health service utilization based on sociodemographic and economic factors in 2006, 2011–12 and 2017 in Lao PDR

Variables	Year 2006 (N = 1622)		Year 2011–12 (N = 4444)		Year 2017 (N = 4460)	
	CIX	Standard errors	CIX	Standard errors	CIX	Standard errors
Use of at least one ANC with skilled personnel						
Mother’s age	− 0.043	0.0181	− 0.014	0.0074	− 0.003	0.0042
Educational attainment	0.313	0.0162	0.222	0.0066	0.101	0.0039
Area of residence	0.281	0.0160	0.158	0.006	0.072	0.0038
Ethnicity	0.227	0.0172	0.162	0.0069	0.082	0.0039
Wealth quintiles	0.330	0.017	0.259	0.0066	0.121	0.0041
Institutional delivery						
Mother’s age	− 0.030	0.0247	− 0.023	0.0099	0.008	0.0058
Educational attainment	0.331	0.0231	0.256	0.0091	0.139	0.0054
Area of residence	0.339	0.0221	0.220	0.008	0.120	0.0052
Ethnicity	0.142	0.0244	0.144	0.0095	0.114	0.0054
Wealth quintiles	0.358	0.0241	0.337	0.0089	0.193	0.0054

Variables	n	Means	Standard deviation	95% CI	P-values^††^	
t-test for comparison of means of CIX absolute values (for year 2017)						
CIX absolute values for ANC	5	0.076	0.045	0.02–0.13	0.011	
CIX absolute values for institutional delivery	5	0.115	0.067	0.03–0.20		

Mean diff of CIX absolute values = mean of CIX absolute values for ANC − mean of CIX absolute values for institutional delivery

*CIX* concentration index, *95% CI* 95% confidence intervals

^††^P-values for one-sided test of CIX (absolute values) mean diff < 0

**Fig. 4:**
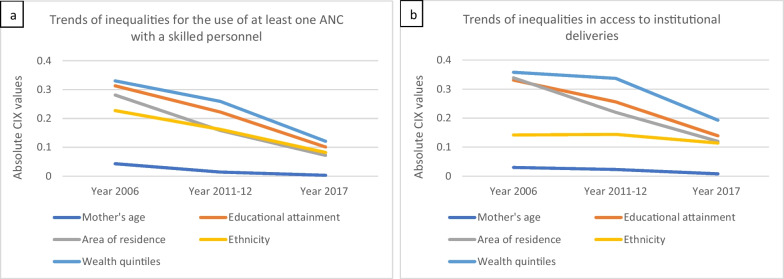
Trends of inequalities in maternal health service utilization between years 2006 and 2017

Figure 4 shows the trends of inequalities in the use of at least one ANC provided by skilled personnel and in institutional delivery by sociodemographic and economic factors between the years 2006 and 2017 in Lao PDR. Between 2006 and 2017, there was a steady decline in the trend of inequalities for all the sociodemographic and economic factors in the use of at least one ANC visit with skilled personnel and institutional delivery. However, for both maternal health services, the most significant reduction of inequalities during the period from 2012 to 2017 was related to wealth quantiles (CIX changed from 0.337 to 0.193 for institutional delivery and from 0.259 to 0.121 for ANC; Table 5). On the other hand, the reduction was the greatest for the area of residence between 2006 and 2012 (CIX changed from 0.339 to 0.220 for institutional delivery and from 0.281 to 0.158 for ANC; Table 5). The rate of reduction remained almost consistent for other factors.

## Discussion

We assessed the trends and magnitude of sociodemographic and economic inequalities in maternal service utilization in the three most recent datasets of Lao PDR. Overall, there was a significant decline in inequality based on the area of residence for the use of maternal health services between 2006 and 2011–12 as CIX changed from 0.281 to 0.158 for ANC compared to 0.339 to 0.220 for institutional delivery. On the other hand, between 2011–12 and 2017, the largest decrease in inequalities was based on wealth quintiles with a CIX change from 0.337 to 0.193 for institutional delivery, and 0.259 to 0.121 for ANC. Despite almost similar inequality levels for all factors except the mother’s age, inequalities remained slightly higher for wealth quintiles in 2017 as CIX were 0.193 and 0.121 for institutional delivery and ANC, respectively.

From 2006 to 2011–12, the middle, and rich quintiles women had the highest increases by 31.7% and 23.3% for ANC use compared to 28.6 and 28% for institutional delivery. Up to 2012, the government of Lao PDR focused on increasing the national rates of basic obstetrics care utilization to achieve the Millennium Development Goals [32]. Efforts were towards improving geographical accessibility and the availability of human resources for health [32]. As a result, although service coverage has improved, it was concentrated among those who may afford services and those living in urban areas with better access to care. Studies conducted before 2012 found that maternal health service utilization was in favor of the rich, those in urban areas, and the women of majority ethnic groups despite national rate increases [22, 33]. In a qualitative study, women from a minority ethnic group in the Nong district reported barriers to service utilization such as discrimination by healthcare providers and lack of acceptability for male providers in caring for pregnant women [33]. Meanwhile, during that period, the largest decline in inequalities was based on the area of residence of women (Table 5).

Between 2011–12 and 2017, the highest increase in ANC was by 37%, 36%, and 31% among the Hmong, women in rural areas with no road access, and the poor, respectively. Whereas the highest increase in institutional deliveries was by 32.8%, 29.3%, and 30.2% in the middle quintile, the poor women and Hmong, respectively. During that period in Lao PDR, several strategies and policies were adopted to enhance the geographic accessibility to and reduce financial barriers to maternal health services in marginalized groups [32, 34]. These actions, including the Free MCH policy adopted early in 2012 align with national and global efforts for Universal Health Coverage [12, 34].

From 2011–12 to 2017, we found that the most significant reduction in inequalities in the use of both maternal health services was wealth related. While it does not directly imply a positive impact of the Free MCH policy by the year 2017, similar studies have shown that user-fee removal policies reduced the gaps in access to ANC and PNC visits [35–39]. The study also revealed that despite the improved service utilization, wealth-related disparities remained the highest in 2017. The gaps between the poorest and richest in 2017, were over 45% and 60% for ANC and institutional deliveries, respectively. Authors suggested that cost subsidization interventions may not be sufficient despite their contribution to reducing financial barriers to service utilization [40]. In Lao PDR, the evaluation of the Free MCH policy’s impact showed that, despite its increase in maternal health service utilization, there was no evidence of pro-poor financial protection [18]. Nonetheless, because the study used data until 2013, a long-term assessment would be appropriate, considering the time needed for behavioral change [41]. Besides, the findings of evaluation may be explained by the “inverse equity hypothesis” which suggests that interventions tend to initially benefit the better-off groups, leading to short-term inequalities due to factors such as unequal geographic distribution of services [18, 42].

The low rate of separate PNC visits, (3.7% in 2017 in Lao PDR despite 47.2% overall PNC coverage) may be attributed to delayed global efforts in promoting PNC services as compared to ANC and facility-based delivery [21, 43–47]. Additionally, the focus on immediate postpartum care might be prioritized due to the higher risks of complications and morbidities for mothers and babies immediately after delivery [48, 49]. Studies also found that women may perceive obstetric care as necessary only in case of complications which could contribute to lower demand for PNC services [50, 51].

### Recommendations

Despite improvements, the coverage gaps between the extreme subgroups of wealth quintiles, education attainment, and area of residence are over 40% for ANC and around 50% for institutional deliveries in the year 2017. Therefore, we recommend investigating and addressing barriers to service utilization specific to each subgroup of women. Key actions may involve providing outreach services to poor women and those in rural areas who may have limited road access to health facilities, understanding and improving women’s perceptions about maternal health services [33, 52–55]. It is important to investigate additional costs that women may incur when seeking services, as reported in earlier studies [18].

### Limitations

Our study has several limitations. Since we did not use regression models to assess inequality, we could not consider possible interactions between factors, and no adjustment for confounders was done [22, 56]. However, the equiplots and the CIX are widely used equity measures including in the Countdown 2015 and 2030 [57]. Also, our analysis may not exactly reflect the current situation in Lao PDR as the latest dataset we used was 5 years old; but it remains the latest nationally representative MICS dataset available.

## Conclusion

Our study revealed that there was a large decline in inequality based on the area of residence for the use of maternal health services between 2006 and 2011–12 while between 2011–12 and 2017, the largest decrease was based on wealth quintiles. Based on evidence from previous studies, it is likely that policies and strategies implemented since 2011–12 such as user-fee removal interventions might have been successful in improving access to maternal health services among the poor in Lao PDR. Meanwhile, more attention should be given to improving the uptake of facility-based PNC visits. Besides, more efforts should be made to further address challenges specific to women subgroups in accessing services and improve financial protection for all women when utilizing services.

## Data Availability

Datasets (MICS 2006, LSIS I 2011–12, and LSIS II 2017) are available in the public domain (https://mics.unicef.org/) and accessible upon request from UNICEF MICS.

## Abbreviations

ANC: Antenatal care
CI: Confidence interval
CIX: Concentration indices
Lao PDR: Lao *People’s Democratic Republic*
LSIS: Lao Social Indicator Survey
MCH: Maternal and Child Health
MICS: Multiple Indicator Cluster Survey
PNC: Postnatal care
WHO: World Health Organization
